# Effects of Forest Bathing on Cardiovascular and Metabolic Parameters in Middle-Aged Males

**DOI:** 10.1155/2016/2587381

**Published:** 2016-07-14

**Authors:** Qing Li, Maiko Kobayashi, Shigeyoshi Kumeda, Toshiya Ochiai, Takashi Miura, Takahide Kagawa, Michiko Imai, Zhiyu Wang, Toshiaki Otsuka, Tomoyuki Kawada

**Affiliations:** ^1^Department of Hygiene and Public Health, Nippon Medical School, 1-1-5 Sendagi, Bunkyo-ku, Tokyo 113-8602, Japan; ^2^Nagano Prefectural Kiso Hospital, 6613-4 Fukushima, Kiso-cho, Kiso-gun, Nagano 397-8555, Japan; ^3^Forest Baubiologie Studio Inc., 3-40-10 Kinugaoka, Hachiouji-shi, Tokyo 192-0912, Japan; ^4^Agematsu Town Office Industry & Tourism Department, 2-13 Ekimae-dori, Agematsu Town, Kiso-gun, Nagano 399-5603, Japan; ^5^Forestry and Forest Products Research Institute, 1 Matsunosato, Tsukuba 305-8687, Japan; ^6^Le Verseau Inc., 3-19-4 Miyasaka, Setagaya-ku, Tokyo 156-0051, Japan; ^7^Clinical Audit Group, Regulatory Affairs Audit Department, Chugai Pharmaceutical Co., Ltd., 1-1 Nihonbashi-Muromachi 2-Chome, Chuo-ku, Tokyo 103-8324, Japan

## Abstract

In the present study, we investigated the effects of a forest bathing on cardiovascular and metabolic parameters. Nineteen middle-aged male subjects were selected after they provided informed consent. These subjects took day trips to a forest park in Agematsu, Nagano Prefecture, and to an urban area of Nagano Prefecture as control in August 2015. On both trips, they walked 2.6 km for 80 min each in the morning and afternoon on Saturdays. Blood and urine were sampled before and after each trip. Cardiovascular and metabolic parameters were measured. Blood pressure and pulse rate were measured during the trips. The Japanese version of the profile of mood states (POMS) test was conducted before, during, and after the trips. Ambient temperature and humidity were monitored during the trips. The forest bathing program significantly reduced pulse rate and significantly increased the score for vigor and decreased the scores for depression, fatigue, anxiety, and confusion. Urinary adrenaline after forest bathing showed a tendency toward decrease. Urinary dopamine after forest bathing was significantly lower than that after urban area walking, suggesting the relaxing effect of the forest bathing. Serum adiponectin after the forest bathing was significantly greater than that after urban area walking.

## 1. Introduction

The forest environment has long been enjoyed for its quiet atmosphere, beautiful scenery, calm climate, pleasant aromas, and clean fresh air. Researchers in Japan have tried to find preventive effects against lifestyle-related diseases from forests and have proposed a new concept called “forest bathing.” What is forest bathing? In Japan, a forest bathing is a short leisurely visit to a forest, called “*Shinrin-yoku*” in Japanese, which is similar in effect to natural aromatherapy, for the purpose of relaxation. “*Shinrin*” means forest and “*yoku*” means bathing in Japanese [1, 2]. Since forests occupy 67% of the land in Japan, forest bathing is easily accessible. Forest bathing as a recognized relaxation and/or stress management activity and a method of preventing diseases and promoting health is becoming a focus of public attention in Japan [2].

We previously found that forest bathing enhances human natural killer (NK) activity by increasing the number of NK cells and intracellular levels of anticancer proteins such as perforin, granulysin, and granzymes in both male and female subjects [1–5]. The increased NK activity was shown to last for more than 30 days after a trip [3, 4]. This has very important implications for preventive medicine. Conversely, taking an urban trip has not been shown to increase human NK activity, numbers of NK cells, or the expression of the selected intracellular perforin, granulysin, and granzymes A/B, indicating that increased NK activity during forest bathing is not due to the trip itself but due to the forest environment [3]. Moreover, forest bathing reduces sympathetic nervous activity and negative emotions, increases parasympathetic nervous activity, and has a relaxing effect on humans [1, 2, 4–9]. Although there have been several studies with healthy young adults as subjects [6–8], few studies have investigated the effects of forest bathing on middle-aged subjects [9]. It is generally accepted that studying the effect of walking in a forest environment on cardiovascular function in middle-aged subjects is more important than that in young male students, and even more so for subjects with higher blood pressure.

Based on the findings mentioned above, because forest environments reduce sympathetic nervous activity and increase parasympathetic nervous activity, we speculated that walking in a forest environment may have beneficial effects on cardiovascular function. Thus, in the present study, we investigated the effects of walking in a forest park on cardiovascular and metabolic parameters in middle-aged males.

## 2. Subjects and Methods

### 2.1. Subjects

In the present study, we investigated the effects of forest bathing on blood pressure and pulse rate during walking by an ambulatory automatic blood pressure monitor and other cardiovascular and metabolic parameters in middle-aged male subjects. Nineteen middle-aged male subjects, ranging in age from 40 to 69 years (mean ± SD: 51.2 ± 8.8), were recruited for the present study (Table 1). Advertisements were placed in newspapers to recruit the subjects with the following conditions: (1) males whose ages are between 40 and 74 years old, (2) people with high-normal or hypertension, and (3) people not taking any antihypertensive drugs. The subjects live and work in small cities. Although the levels of systolic and diastolic blood pressure were 144.0 ± 9.9 mmHg and 92.6 ± 7.4 mmHg, respectively, these subjects were not taking any antihypertensive drugs. Information about the subjects was gathered from a self-administered questionnaire that asked about cigarette smoking, alcohol consumption, and sleeping hours and has been reported previously [9, 10]. Written informed consent was obtained from all subjects after a full explanation of the study procedures. None of the subjects had any symptoms of disease, used drugs that might have affected the results, or were taking any medication at the time of the study. The subjects took the same breakfast and lunch during the two trips. To control for the effects of alcohol, the subjects did not consume alcohol during the study period. The Ethics Committees of the Nippon Medical School and Nagano Prefectural Kiso Hospital approved this study.

### 2.2. Walking in a Forest Environment and in an Urban Area

**Figure 1:**
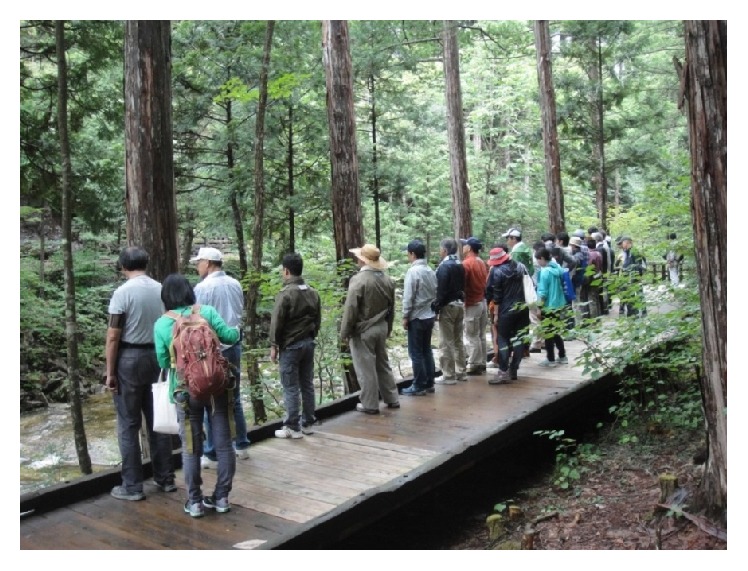
Forest bathing.

**Figure 2:**
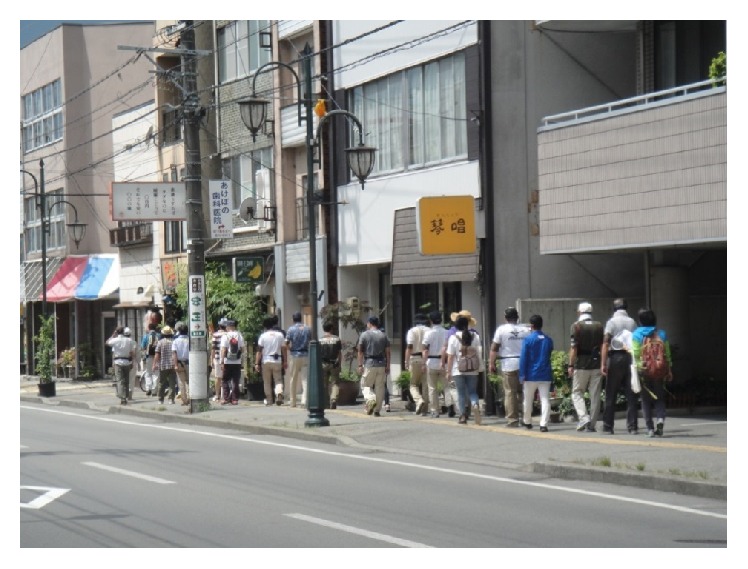
Urban area walking.

We previously found that the effects of walking in forest environments on the immune function (natural killer activity) lasted for more than one week, and sometimes even 4 weeks, but not walking in urban environments [3–5]. Therefore, to avoid such lasting effects, we designed the study so that all subjects first walked in the urban area and then in the forest. The interval between the two experiments was one week. The subjects took day trips to a forest park named Akasawa Shizen Kyuyourin (Akasawa Natural Recreation Forest) in Agematsu, Nagano Prefecture (situated in central Japan) (Figure 1) on August 29, 2015, and to an urban area of Nagano Prefecture where there were almost no trees as a control (Figure 2) on August 22, 2015. On both trips, they walked 2.6 km for 80 min with the same speed guided by the some guide in the morning (11:00–12:20) and afternoon (13:40–15:00) on Saturdays. The subjects did not communicate with each other during the walk to avoid the effects of talking. Both waking courses are the flat walking ways without any slope. To control for the effects of cigarette smoking, the smokers did not smoke during the walking. To control for the effects of caffeine, the subjects were only allowed to drink mineral water during the walking.

### 2.3. Physiological and Psychological Indices

Blood and urine were sampled in the morning before and after each trip. Cardiovascular and metabolic parameters were measured. Blood pressure and pulse rate were measured by an ambulatory automatic blood pressure monitor at the same time every 20 min during each trip. The Japanese version of the profile of mood states (POMS) test was conducted before, during, and after the trips [1]. Ambient temperature and humidity were monitored during the trips.

### 2.4. Blood Analysis [9]

The serum levels of triglycerides, total cholesterol (Cho), low density lipoprotein (LDL) Cho, high density lipoprotein (HDL) Cho, and remnant-like particles (RLP) Cho were analyzed using an enzymatic assay with an autoanalyzer. The serum total adiponectin concentration was measured using an enzyme immunoassay (EIA). Blood glucose concentration was analyzed using a Glucocard GT-1640 and Diasenser strips (Arkray, Kyoto, Japan). The serum level of insulin was analyzed using a chemiluminescent immunometric assay (CLIA), and the serum level of dehydroepiandrosterone sulfate (DHEA-S) was analyzed using a chemiluminescence enzyme immunoassay (CLEIA). The serum level of high-sensitivity C-reactive protein (hs-CRP) was analyzed using a latex nephelometric assay.

### 2.5. Urinary Adrenaline, Noradrenaline, and Dopamine Measurements

The levels of adrenaline, noradrenaline, and dopamine in urine were measured by an HPLC method using an HLC-725CAII analyzer. The instrument features a column-switching system composed of two pretreatment columns, a separation column, and a high-sensitivity detection unit based on a postcolumn reaction using the fluorogenic reagent 1,2-diphenylethylenediamine. The detection limits of adrenaline, noradrenaline, and dopamine in urine were all 8 fmol/mL [3–5]. The values of urinary adrenaline, noradrenaline, and dopamine were corrected by urinary creatinine and indicated as *μ*g/g creatinine.

### 2.6. Statistical Analysis

The paired *t*-test was used to compare the differences between urban and forest environments. The differences between before and after the walking in some results were also compared by the paired *t*-test. The analyses were performed with the Microsoft Excel software package for Windows. The significance level for *p* values was set at 0.05.

## 3. Results

It was sunny weather in the urban trip, whereas it was rainy weather in the morning and was cloudy in the afternoon in the forest bathing. The respective maximum and average temperatures and average humidity were 32.7°C, 31.2 ± 0.7°C, and 52.4 ± 2.6% during the morning and 37.5°C, 33.2 ± 1.4°C, and 47.5 ± 4.3% during the afternoon in the urban area environment, whereas in the forest environment, the maximum and average temperatures and average humidity were 20.4°C, 19.1 ± 0.5°C, and 94.3 ± 3.9% during the morning and 20.7°C, 19.4 ± 0.4°C, and 90.5 ± 4.2% during the afternoon, respectively.

As show in Table 1, the cohort demographics of 19 middle-aged male subjects are as follows: (1) the average of age was 51.2 ± 8.8 years (mean ± SD), (2) the levels of average systolic and diastolic blood pressure were 144.0 ± 9.9 mmHg and 92.6 ± 7.4 mmHg, respectively, (3) the average of pulse rate was 73.4 ± 12.1 bpm, (4) the average of height was 173.7 ± 6.1 cm, (5) the average of body weight was 71.2 ± 11.7 kg, (6) the average of BMI was 23.7 ± 3.7, and (7) five subjects were smokers and 14 subjects were nonsmokers. These subjects were not taking any antihypertensive drugs.

### 3.1. Effects of Forest Bathing on Pulse Rate

**Figure 3:**
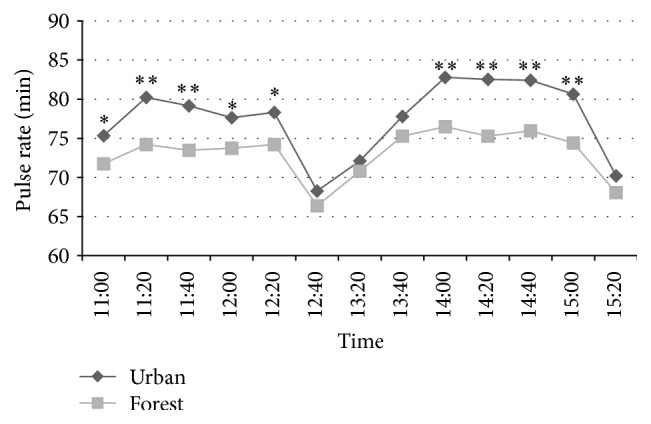
Forest bathing program significantly reduces the pulse rate in male subjects. ^*∗*^ *p* < 0.05, ^*∗∗*^ *p* < 0.01, forest versus urban by paired *t*-test (*n* = 19).

As shown in Figure 3, forest bathing significantly reduced the subjects' pulse rate during 11:00–12:20 and 14:00–15:00, suggesting the relaxing effect of this program.

### 3.2. Effects of Forest Bathing on Feelings in POMS Test

**Figure 4:**
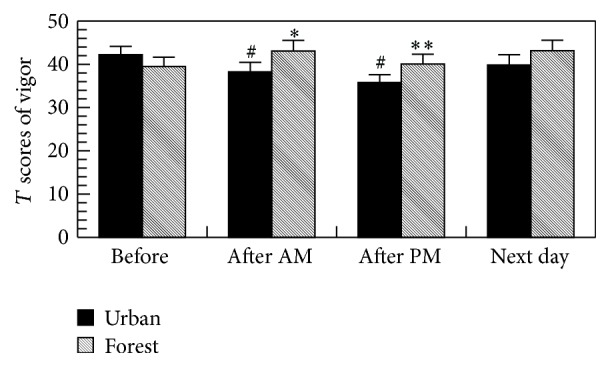
Effects of forest bathing on the *T* scores of vigor in the POMS test. ^*∗*^ *p* < 0.05, ^*∗∗*^ *p* < 0.01, versus urban, and ^#^ *p* < 0.01 versus before by paired *t*-test (mean + SE, *n* = 19); after AM: after walking on the morning and after PM: after walking on the afternoon.

As shown in Figure 4, the forest bathing significantly increased the score for vigor, whereas urban area walking significantly decreased the score for vigor in the POMS test, suggesting the relaxing effect of forest bathing program.

**Figure 5:**
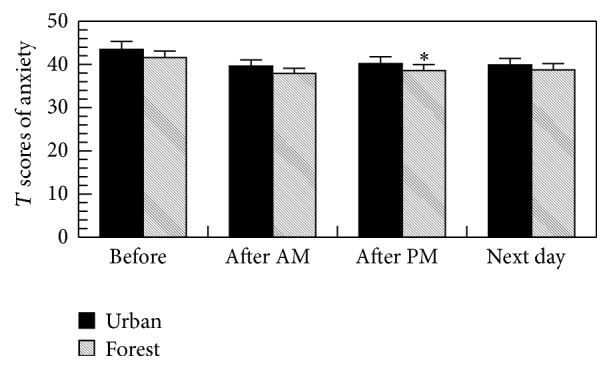
Effects of forest bathing on the *T* scores of anxiety in the POMS test. ^*∗*^ *p* < 0.05 versus urban, by paired *t*-test (mean + SE, *n* = 19); after AM: after walking on the morning and after PM: after walking on the afternoon.

As shown in Figure 5, there was a significant decrease in the scores for anxiety in the POMS test after walking in the forest on the afternoon compared to that walking in the urban, suggesting the relaxing effect of forest bathing program.

**Figure 6:**
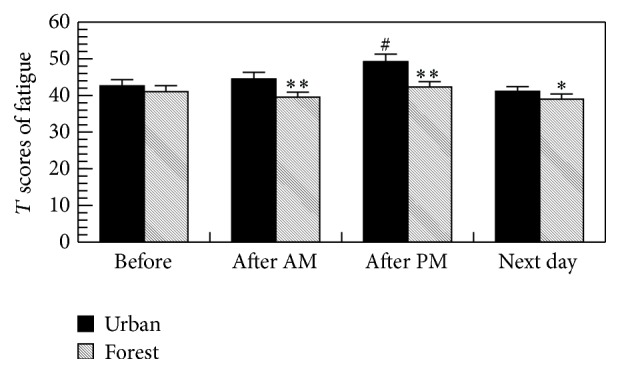
Effects of forest bathing on the *T* scores of fatigue in the POMS test. ^*∗*^ *p* < 0.05, ^*∗∗*^ *p* < 0.01, versus urban, ^#^ *p* < 0.01 versus before by paired *t*-test (mean + SE, *n* = 19); after AM: after walking on the morning and after PM: after walking on the afternoon.

As shown in Figure 6, the forest bathing significantly decreased the scores for fatigue, whereas urban area walking significantly increased the score for fatigue in the POMS test, suggesting the relaxing effect of forest bathing program.

**Figure 7:**
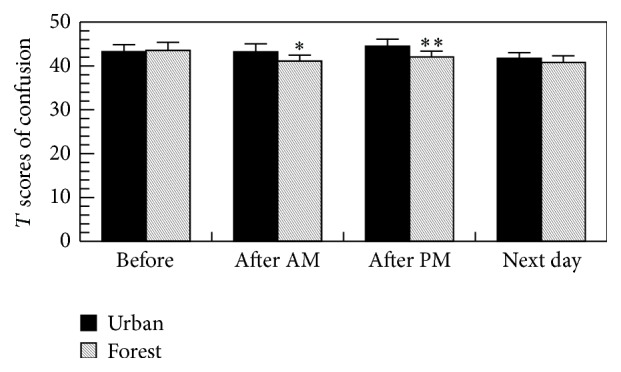
Effects of forest bathing on the *T* scores of confusion in the POMS test. ^*∗*^ *p* < 0.05, ^*∗∗*^ *p* < 0.01, versus urban by paired *t*-test (mean + SE, *n* = 19); after AM: after walking on the morning and after PM: after walking on the afternoon.

As shown in Figure 7, the forest bathing significantly decreased the scores for confusion in the POMS test, suggesting the relaxing effect of forest bathing program.

There was a significant decrease in the scores for depression in the POMS test after walking in the forest on the morning compared to that before the walking, suggesting the relaxing effect of forest bathing program (data not shown). Both trips did not affect the scores for angry (data not shown).

### 3.3. Effects of Forest Bathing on Urinary Adrenaline, Noradrenaline, and Dopamine

As shown in Table 2, both trips significantly reduced the level of urinary noradrenaline. Although there was no significant difference between before and after forest walking, the urinary adrenaline level showed a tendency toward decrease after forest walking. The urinary dopamine level after forest bathing was significantly lower than that after urban area walking; however, there was no difference in baseline (before the trips), suggesting that forest bathing may have a beneficial effect on urinary dopamine.

### 3.4. Effects of Forest Bathing on the Level of Adiponectin in Serum

**Figure 8:**
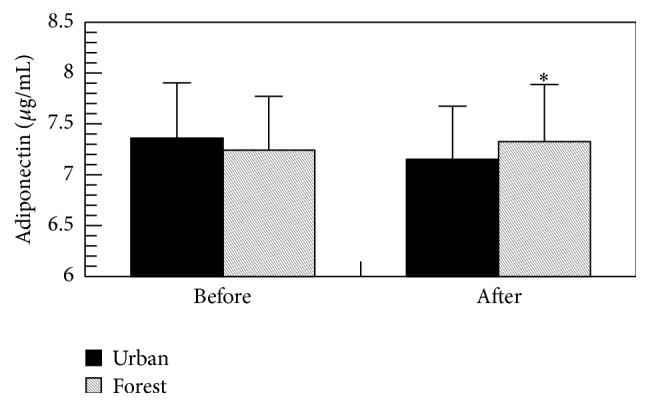
Effect of forest bathing program on the level of adiponectin in male subjects. ^*∗*^ *p* < 0.05, versus urban by paired *t*-test (mean + SE, *n* = 19).

As shown in Figure 8, the level of adiponectin in serum after forest bathing was significantly greater than that after urban area walking; however, there was no difference in baseline (before the trips), suggesting that forest bathing may have a beneficial effect on adiponectin in serum.

### 3.5. Effects of Forest Bathing on Blood Pressure

**Figure 9:**
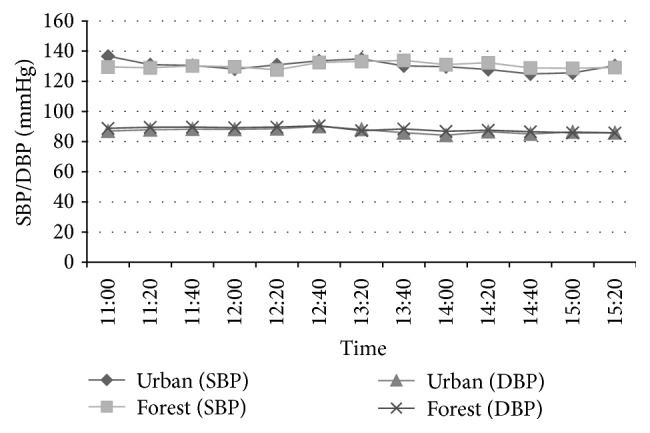
Effect of forest bathing on blood pressure level in male subjects (*n* = 19).

As shown in Figure 9, there was no significant difference in blood pressure between forest and urban area walking during the trips because of the big difference in ambient temperature between the forest (lower temperature) and urban area (higher temperature) environments.

### 3.6. Effects of Forest Bathing on Metabolic Parameters

Neither walking in the forest nor walking in the urban area affected the levels of triglycerides, total cholesterol (Cho), low density lipoprotein Cho, high density lipoprotein Cho, insulin, HbA1c, or high-sensitivity C-reactive protein in serum, or blood glucose. Both trips also had no effect on the numbers of white blood cells, red blood cells, and platelets, lymphocytes, granulocytes, or monocytes or the Hb concentration in the peripheral blood (data not shown).

## 4. Discussion

It is generally accepted that studying the effect of walking in forest environments on cardiovascular function in middle-aged subjects is more important than that in young male students, especially in subjects with a higher blood pressure. However, few studies have investigated the effects of forest bathing on middle-aged hypertensive subjects [9]. Thus, in the present study we evaluated the effects of forest bathing on cardiovascular function in middle-aged hypertensive subjects.

We found that walking in a forest park significantly reduced the pulse rate in middle-aged males with higher blood pressure, compared with walking in an urban area. Because pulse rate is a basic index of activity of the autonomic nervous system, this decrease in pulse rate indicates a state of relaxation in the subjects. We previously also found that a forest bathing program significantly reduced the pulse rate in both middle-aged males [12] and females [13].

The forest bathing significantly increased the score for vigor and decreased the scores for depression, anxiety, fatigue, and confusion in the POMS test, whereas urban area walking significantly increased the score for fatigue and decreased the score for vigor, suggesting a relaxing effect of the forest bathing program. We previously also found that a forest bathing program significantly reduced the scores for depression, fatigue, anxiety, angry, and confusion and increased the score for vigor in the POMS test in both males [1, 2, 5–9] and females [2, 4, 13].

Why does forest bathing reduce the pulse rate and the scores for depression, fatigue, and confusion in the POMS test? To answer this question, we measured the levels of urinary adrenaline, noradrenaline, and dopamine in the present study. Although there was no significant difference between before and after forest walking, the urinary adrenaline level showed a tendency toward decrease after forest walking. The level of urinary dopamine after forest bathing was significantly lower than those after urban walking. It has been reported that sympathetic nerve activity can be determined by measuring the levels of urinary adrenaline, noradrenaline, and/or dopamine [14], suggesting that sympathetic nerve activity was lower during forest bathing. We previously found that forest bathing significantly reduced the levels of urinary adrenaline and noradrenaline in both male and female subjects [3–5, 9].

The level of adiponectin in serum after forest bathing was significantly greater than that after urban area walking; however, there was no difference in baseline (before the trips), suggesting that forest bathing may have a beneficial effect on adiponectin in serum. Adiponectin is a serum protein hormone that is specifically produced by adipose tissue. Studies have shown that lower-than-normal blood adiponectin concentrations are associated with several metabolic disorders, including obesity, type 2 diabetes mellitus, cardiovascular disease, and metabolic syndrome [15]. This result supports our previous finding that forest bathing significantly increased the serum adiponectin level in middle-aged males [9].

There was no significant difference in blood pressure between the forest and urban area walking during the trips because of the big difference in ambient temperature between the forest (lower temperature) and urban (higher temperature) environments. It has been reported that a higher ambient temperature reduces blood pressure, whereas a lower ambient temperature raises blood pressure [16–19]. Moreover, Woodhouse et al. [16] reported that the blood pressure of elderly people may be inversely related to the ambient temperature and that after adjustment for confounding seasonal effects, a 1°C decrease in living-room temperature was associated with rises of 1.3 mmHg in SBP and 0.6 mmHg in DBP. Hozawa et al. [18] also reported that when the outside temperature was ≥10°C, a 1°C increment of outside temperature corresponds to 0.4 mmHg and 0.28 mmHg decrease of SBP and DBP. Based on these findings, the blood pressure levels of subjects should have been higher in forest than in urban area because of the lower temperature in the forest; however, the blood pressure levels of subjects in the forest were almost the same as those in the urban area, suggesting that forest environment prevented the increase in blood pressure that should have been seen due to the lower temperature. In other words, the forest bathing contributed to the control of blood pressure and had a beneficial effect on blood pressure. Further studies should be conducted with the same ambient temperatures in forest and urban areas. In fact, we previously found that forest bathing significantly reduced blood pressure by reducing sympathetic nerve activity and urinary adrenaline, and noradrenaline and dopamine levels; in that study the ambient temperature in the forest was almost the same as that in the urban area [9].

Neither walking in the forest nor walking in the urban area affected the levels of triglycerides, total cholesterol (Cho), low density lipoprotein Cho, high density lipoprotein Cho, insulin, HbA1c, or high-sensitivity C-reactive protein in serum, or blood glucose. Both trips also had no effect on the numbers of white blood cells, red blood cells, and platelets, lymphocytes, granulocytes, or monocytes or the Hb concentration in the peripheral blood (data not shown), which are similar to the previous study [9].

We previously found that the effects of walking in forest environments on the immune function (natural killer activity) lasted for more than one week, but not walking in urban environments [3–5]. Therefore, to avoid such lasting effects, we designed the study so that all subjects first walked in the urban area and then in the forest in the present study. Because there were no carry-over effects when subjects walked in an urban environment [3] the interval between the two experiments was one week.

The subjects did not communicate with each other during the walk to avoid the effects of talking. Therefore, although all participants completed the walks together it would not affect the results. However, the order of exposure (forest versus urban) is not counterbalanced and all participants completed the urban walk followed by the forest walk to avoid any carry-over effects after forest bathing. This is a limitation of the present study.

It was very difficult to recruit the subjects in this study. We placed advertisements in newspapers to recruit the subjects. Maybe they are not representative of the broader population and an ideal recruitment of subjects would use a randomized stratification. This is another limitation of the present study.

## 5. Conclusions

Despite the bad weather conditions (rainy weather and lower ambient temperature) during the forest bathing, our study indicated that forest bathing produced the following significant benefits compared to urban area walking:

1. Decrease in pulse rate.
2. Decrease in urinary dopamine.
3. Tendency toward decrease in urinary adrenaline.
4. Increase in adiponectin in serum.
5. Decreases in negative moods such as anxiety, depression, fatigue, and confusion.
6. Increase in feelings of vigor in the POMS test in middle-aged males with higher blood pressure.

Taken together, the forest bathing program induced significant physiological and psychological relaxation. These findings clarified the physiological and psychological effects of the forest bathing program and suggested a possibility of clinical use. We recommend conducting forest bathing in a warmer day.

## Figures and Tables

**Table 1:** Information of the subjects.

Number	Age (year)	Height (cm)	Body weight (kg)	BMI	SBP (mmHg)	DBP (mmHg)	Pulse rate/min	Remark^#^	Smoking status
1	69	168	57	20	142	83	63	Stage I HT	No
2	67	179	72	22	138	85	60	High-normal	No
3	40	184	87	26	127	81	92	Normal	Smoking
4	56	176	62	20	148	84	101	Stage I HT	No
5	44	166	74	29	144	93	73	Stage I HT	No
6	46	180	68	21	143	92	79	Stage I HT	Smoking
7	55	167	54	19	157	103	81	Stage II HT	No
8	49	173	65	22	150	99	62	Stage I HT	No
9	40	179	84	26	146	99	68	Stage I HT	No
10	44	172	74	25	148	92	80	Stage I HT	Smoking
11	46	184	70	21	143	84	96	Stage I HT	Smoking
12	46	165	58	21	147	98	70	Stage I HT	No
13	49	172	78	26	149	104	79	Stage I HT	No
14	66	172	70	23	161	95	61	Stage II HT	No
15	50	170	63	22	155	106	64	Stage II HT	Smoking
16	44	170	102	35	131	93	75	Stage I HT	No
17	45	165	61	22	141	84	60	Stage I HT	No
18	59	176	70	23	140	90	64	Stage I HT	No
19	58	182	85	26	126	94	67	Stage I HT	No

Mean	51.2	173.7	71.2	23.7	144.0	92.6	73.4		
SD	8.8	6.1	11.7	3.7	9.0	7.4	12.1		
SE	2.0	1.4	2.7	0.9	2.1	1.7	2.8		

^#^Based on the Japanese Society of Hypertension Guidelines [11]. HT: hypertension.

**Table 2:** Effect of forest bathing on the levels of urinary noradrenaline, dopamine, and adrenaline in male subjects.

	Before walking	After walking
*Noradrenaline*		
Urban	93.17 ± 6.57	78.46 ± 5.56^##^
Forest	79.35 ± 5.95^*∗*^	70.29 ± 6.36^*∗*,#^

*Dopamine*		
Urban	503.65 ± 31.23	560.40 ± 39.25
Forest	428.66 ± 28.59	447.97 ± 20.76^*∗∗*^

*Adrenaline*		
Urban	5.50 ± 0.84	5.371 ± 1.09
Forest	5.04 ± 0.73	4.42 ± 0.63

Mean ± SE, *n* = 19, ^*∗*^ *p* < 0.05, ^*∗∗*^ *p* < 0.01 versus urban; ^#^ *p* < 0.05, ^##^ *p* < 0.01 versus before, by paired *t*-test.
